# Effects of *Bacillus amyloliquefaciens* LFB112 on Growth Performance, Carcass Traits, Immune, and Serum Biochemical Response in Broiler Chickens

**DOI:** 10.3390/antibiotics10111427

**Published:** 2021-11-22

**Authors:** Marhaba Ahmat, Junhao Cheng, Zaheer Abbas, Qiang Cheng, Zhen Fan, Baseer Ahmad, Min Hou, Ghenijan Osman, Henan Guo, Junyong Wang, Rijun Zhang

**Affiliations:** 1 Laboratory of Feed Biotechnology, State Key Laboratory of Animal Nutrition, College of Animal Science and Technology, China Agricultural University, Beijing 100193, China; malika511@126.com (M.A.); chengjunhao@cau.edu.cn (J.C.); Zaheerabbas113@yahoo.com (Z.A.); chengqiangcool@163.com (Q.C.); ghn_657@cau.edu.cn (H.G.); wangjy9722@163.com (J.W.); 2 Xinjiang Laboratory of Special Environmental Microbiology, Institute of Applied Microbiology, Xinjiang Academy of Agricultural Sciences, Urumqi 830091, China; hmde_092@163.com (M.H.); ghenijan85@foxmail.com (G.O.); 3 TECON Biology Company, LTD 528 Changchun South Road, Urumqi 830091, China; fanzhen1222@outlook.com; 4 Faculty of Veterinary and Animal Sciences, Muhammad Nawaz Sharif University of Agriculture, Multan 25000, Pakistan; dr.baseerahmadkhan@gmail.com

**Keywords:** *Bacillus amyloliquefaciens*, broiler chickens, growth performance, carcass traits, immunity, serum chemical parameters

## Abstract

This study aimed to investigate the effects of *Bacillus amyloliquefaciens* LFB112 on the growth performance, carcass traits, immune response, and serum biochemical parameters of broiler chickens. A total of 396 1 day old, mixed-sex commercial Ross 308 broilers with similar body weights were allotted into six treatment groups. The assigned groups were the CON group (basal diet with no supplement), AB (antibiotics) group (basal diet + 150 mg of aureomycin/kg), C+M group (basal diet + 5 × 10^8^ CFU/kg *B. amyloliquefaciens* LFB112 powder with vegetative cells + metabolites), C group (basal diet + 5 × 10^8^ CFU/kg *B. amyloliquefaciens* LFB112 vegetative cell powder with removed metabolites), M group (basal diet + 5 × 10^8^ CFU/kg *B. amyloliquefaciens* LFB112 metabolite powder with removed vegetative cells), and CICC group (basal diet + 5 × 10^8^ CFU/kg *Bacillus subtilis* CICC 20179). Results indicated that chickens in the C+M, C, and M groups had higher body weight (BW) and average daily gain (ADG) (*p* < 0.05) and lower feed conversion ratio (FCR) (*p* = 0.02) compared to the CON group. The C+M group showed the lowest abdominal fat rate compared to those in the CON, AB, and CICC groups (*p* < 0.05). Compared to the CON group, serum IgA and IgG levels in the C+M, C, and M groups significantly increased while declining in the AB group (*p* < 0.05). *B. amyloliquefaciens* LFB112 supplementation significantly reduced the serum triglyceride, cholesterol, urea, and creatinine levels, while increasing the serum glucose and total protein (*p* < 0.05). In conclusion, *B. amyloliquefaciens* LFB112 significantly improved the growth performance, carcass traits, immunity, and blood chemical indices of broiler chickens and may be used as an efficient broiler feed supplement.

## 1. Introduction

Dietary antibiotics have brought great benefits to the development of the livestock and poultry industry for more than 60 years. They have been used to improve growth performance by controlling the growth and proliferation of pathogenic microorganisms in the gastrointestinal tract, resulting in better digestion, absorption, and metabolism of nutrients [1,2]. Although the application of antibiotics shows some affirmative effect on poultry production, continuous consumption results in problems such as the development of cross-resistance and multi-antibiotic resistance in pathogenic bacteria [3], drug residues in the final product, and dysbiosis [4]. Antibiotic residues in animal products are essentially a double-edged sword as they threaten human health, as well as lead to possible antibiotic-resistant pathogenic bacteria and intestinal flora disorders [5,6]. In response to this situation, growth-promoting antibiotics have been prohibited as feed supplements in Europe since 2006, in the United States since 2014, and in China since 2020. Consequently, pursuing safe, effective, and non-residual antibiotic alternatives has become the focus of the animal husbandry industry. Various studies have confirmed numerous biological substances such as probiotics, plant extracts, essential oils, antimicrobial peptides, acidifiers, and enzymes as potential substitutes for antibiotics [5,6]. Among them, probiotics are considered ideal substitutes for antibiotics due to their nontoxic effects, lack of residue, and significant effects on animal health and production.

Probiotics are live microbes that, when administered in a suitable amount, exert beneficial effects on the host [7,8]. In particular, the intestinal flora as regulable probiotics is an effective substitute for antibiotics. Various studies have claimed that probiotics positively affect intestinal morphology, intestinal microbial population, energy utilization, body antioxidant regulation, and immune response, thus favoring broiler gut health and production performance [9,10,11]. Due to their beneficial effects, several probiotics have been applied in the poultry industry, including *Lactobacillus, Bacillus, Bifidobacterium, Streptococcus, Enterococcus, Aspergillus, Candida*, and *Saccharomyces* strains [12]. Among them, spore-forming *Bacillus* species are expanding rapidly with a growing number of studies demonstrating growth promotion, immunomodulation, and competitive exclusion, as their spores are heat-stable, as well as acid- and bile-salt-resistant in the gastrointestinal tract [13,14]. Moreover, the production of a wide range of extracellular substances and antimicrobial peptides against a variety of pathogens has been confirmed in *Bacillus* species [15].

*Bacillus subtilis*, one of the most well-researched probiotics, has been widely used as a direct-fed microbial (DFM) feed supplement [16,17], thus improving feed digestion and utilization via regulating intestinal flora [18]. Furthermore, it enhances immunity [19] and the growth performance of the animal via secretion of various digestive enzymes [20]. *Bacillus subtilis* is heat- and acid-resistant, and it is a facultative anaerobe that can grow in the gut [21]. *Bacillus subtilis* was previously considered a strictly aerobic bacterium, but it was later found to propagate anaerobically in the presence of nitrate [22]. The anaerobic property is controlled by two gene regulatory proteins, ResD and ResE [23], which consume free oxygen in the intestinal tract, inhibiting the growth of harmful aerobic bacteria. By doing so, the beneficial anaerobic bacteria become the dominant flora in the gut, which explains how *Bacillus subtilis* can play an important role in an intestinal anaerobic environment. Previous studies have indicated that *B. subtilis* can enhance heat-stressed broiler growth performance, as well as the recovery and restoration processes of damaged intestinal mucosa [24,25], contend with pathogens, adjust intestinal microbiota, and promote resilience in chickens [26,27].

*Bacillus amyloliquefaciens* is a vigorous *Bacillus* species that releases extracellular enzymes, such as *β*-amylases, cellulase, metalloproteases, and proteases that can improve digestion, nutrient absorption, and gut resistance to infection [28,29]. Previously, *Bacillus amyloliquefaciens* LFB112 was reported to secrete bacteriocins with good thermostability and acid/alkali tolerance. These bacteriocins were effective against a wide range of pathogens, including both Gram-positive and Gram-negative bacteria involved in various animal diseases [30,31]. In addition, our group has demonstrated that *Bacillus amyloliquefaciens* LFB112 significantly improves growth performance, meat quality, and gut health in broilers [32]. Nevertheless, to our knowledge, information is lacking about the effects of *Bacillus amyloliquefaciens* LFB112 on carcass trait, immunity, and blood chemical parameters of broiler chickens. Furthermore, we hypothesized that, along with living bacteria LFB112 (pure-spore bacteria), its cell-free metabolites have beneficial effects on broiler chickens. Therefore, this study was designed to assess the effectiveness of *Bacillus amyloliquefaciens* LFB112 (vegetative cells + metabolites) and its cell-free metabolites on the growth performance, carcass trait, immune response, and serum biochemical parameters of broiler chickens. This will provide a scientific basis for the application of antibiotic alternatives.

## 2. Results

### 2.1. Growth Performance

The feed intake, body weight gain, and feed conversion ratio of broilers during the experimental period are summarized in Table 1. From day 1 to 21, birds in the C + M, C, and M groups had higher ADG than those in the CON and AB groups (*p* < 0.05); there were no significant differences (*p* > 0.05) among all groups in average daily feed intake (ADFI). However, the C group had the lowest FCR compared to the other groups (*p* < 0.01). From day 22 to 39, the C + M, C, and M groups had higher (*p* < 0.01) ADG and BW, while the control group showed the lowest ADG, with no significant difference when comparing the control to the AB and CICC groups. Moreover, the C + M group had a significantly lower FCR (*p* = 0.02) than the control and other treatment groups during this period. Over the whole study period (day 0 to 39), the *B. amyloliquefaciens* LFB112-supplemented groups had higher ADG and ADFI (*p* < 0.001) than the CON group and other dietary treatments. Broilers in the C group had greater ADFI (*p* < 0.05) than those in the control and other groups. Furthermore, the C + M group showed the lowest FCR (1.60) (*p* < 0.05), compared to the other groups, followed by the AB group (1.63), and the highest rate of FCR was obtained by the CON and C groups (1.65), but did not significantly differ from the AB, M, and CICC groups. In general, the supplementation of LFB112 or its metabolites (C + M, C, and M groups) significantly increased (*p* < 0.001) the final BW and total weight gain of broilers compared with normal and antibiotic-supplemented diets.

### 2.2. Carcass Traits

In Table 2, details on carcass characteristics are presented at day 39. Broilers in the C + M and C groups had higher carcass yield, semi-eviscerated rate, eviscerated rate, thigh muscle yield, and breast muscle yield than CON and other groups (*p* ≤ 0.01). In particular, the carcass yield, semi-eviscerated rate, and eviscerated rate of broilers in the C + M group were on average 3.54%, 6.05%, and 5.69% higher (*p* < 0.05) than those in the control group, respectively. Additionally, the C + M group showed the lowest abdominal fat rate of 1.21%, which was on average 0.51%, 0.16%, and 0.37% lower (*p* < 0.05) than in the CON, AB, and CICC groups, respectively. Dietary *B. amyloliquefaciens* LFB112-based supplements significantly improved the carcass yield and reduced broilers chicken abdominal fat (*p* < 0.05).

### 2.3. Immune Organs and Serum Immune Factors

**Figure 1:**
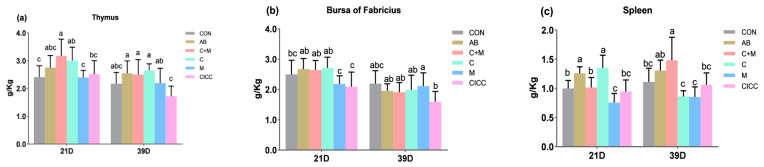
Effect of *Bacillus amyloliquefaciens* LFB112 on immune organ index (g/kg) of broilers. (**a**) Effect of *Bacillus amyloliquefaciens* LFB112 on thymus. (**b**) Effect of *Bacillus amyloliquefaciens* LFB112 on bursa of Fabricius. (**c**) Effect of *Bacillus amyloliquefaciens* LFB112 on spleen. Experimental groups assigned were as follows: CON = basal diet; AB (antibiotics) = basal diet + aureomycin 150 mg/kg; C + M = basal diet + LFB112 fermentation dry powder with *Bacillus* cells + metabolites (5 × 10^8^ CFU/g); C = basal diet + LFB112 *Bacillus* cells powder with removed metabolites (5 × 10^8^ CFU/g); M = basal diet + LFB112 metabolite powder with removed *Bacillus* cells (5 × 10^8^ CFU/g); CICC = basal diet + *Bacillus subtilis* 20,179 (5 × 10^8^ CFU/g). (**a**–**c**) Different letters on standard error bars indicate a significant difference (*p* < 0.05). Data are shown as means and standard errors (*n* = 6).

On day 21, the thymus index of broilers in C + M and C groups increased by 30.99% and 24.79%, respectively (*p* < 0.05), while that in the AB group increased by 13.64% compared to the control group (Figure 1, Supplementary Table S1). However, there were no significant differences among the CON, C, and CICC groups. The bursa index of M and CICC groups was lower (*p* < 0.05) than that of the control and other treatment groups, but there was no significant difference in the bursa index between the CON and other groups. The spleen index in AB and C groups was increased (*p* < 0.05), while that in M and CICC groups was decreased (*p* < 0.05) as compared to the CON and C + M groups. Broiler chickens fed with CICC showed a lower thymus index and bursa index at day 39 (*p* < 0.05), but there were no significant differences among the other treatment groups. The spleen index of the C + M group increased by 34.5%, 12.12%, 52.6%, 59.2%, and 38.3% compared with the CON, AB, C, and CICC groups at day 39 (*p* < 0.001), respectively.

**Figure 2:**
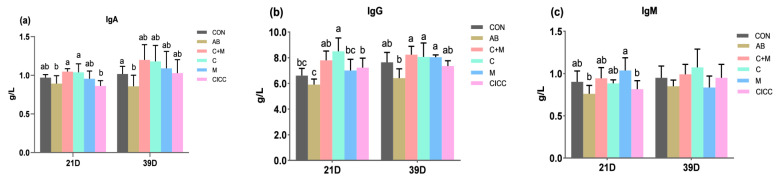
Effect of *Bacillus amyloliquefaciens* LFB112 on serum immune factors (g/L) of broiler. Ig = immunoglobulin. (**a**) Effect of *Bacillus amyloliquefaciens* LFB112 on IgA. (**b**) Effect of *Bacillus amyloliquefaciens* LFB112 on IgG. (**c**) Effect of *Bacillus amyloliquefaciens* LFB112 on IgM. Experimental groups assigned were as follows: CON = basal diet; AB (antibiotics) = basal diet + aureomycin 150 mg/kg; C + M = basal diet + LFB112 fermentation dry powder with *Bacillus* cells + metabolites (5 × 10^8^ CFU/g); C = basal diet + LFB112 *Bacillus* cell powder with removed metabolites (5 × 10^8^ CFU/g); M = basal diet + LFB112 metabolite powder with removed *Bacillus* cells (5 × 10^8^ CFU/g); CICC = basal diet + *Bacillus subtilis* 20,179 (5 × 10^8^ CFU/g). (**a**–**c**) Different letters on standard error bars indicate a significant difference (*p* < 0.05). Data are shown as means and standard errors (*n* = 6).

As reported in Figure 2 and Supplementary Table S2, at day 21, compared to the control group, the serum immune index of IgA in AB and CICC groups decreased by 8.3% and 11.3% (*p* < 0.05), respectively. Those in C + M and C groups increased by 8.3% and 7.2%, respectively; however, there was no significant difference as compared with CON (*p* > 0.05). The addition of LFB112 fermentation powder (C + M) and metabolites (M) significantly increased (*p* < 0.05) serum IgG level, while other groups had no significant effect on the IgG content. The IgM of serum showed an increasing trend in all treatments (*p* < 0.05). Lower values were observed in AB and CICC groups (*p* > 0.05), which were statistically similar to each other, whereas higher values were observed in other treatments (*p* > 0.05), which did not differ from each other. Serum IgA and IgG of broilers at day 39 that received LFB112 (C + M, C, and M) showed a significant increase (*p* > 0.05) by 17.6%, 15.7%, and 6.7% and by 7.7%, 5.5%, and 5.1%, respectively, compared with the control; however, no significant differences were observed among the three groups. Dietary supplementation of AB also decreased IgA and IgG content in the serum of broilers (*p* < 0.05). No significant difference was detected in serum IgM content among all dietary treatments.

### 2.4. Serum Biochemical Parameters

The blood serum biochemical parameters of the experimental groups are given in Table 3. On day 21, the serum glucose showed a significant increase among C + M, C, and M groups (*p* > 0.05), but no significant differences compared to the CON group. The addition of C + M, C, and M to the diet increased the content of serum glucose in birds at day 21 (*p* > 0.05), but no significant differences were observed compared with the control. The glucose content of the CICC group was lower (*p* < 0.05) than others, with no significant difference compared to the CON group. Dietary AB significantly increased (*p* < 0.05) the contents of serum total triglyceride, cholesterol, urea, and creatinine. At the same time, the addition of *B. amyloliquefaciens* LFB112 and its metabolites could reduce (*p* > 0.05) the contents of serum triglyceride, cholesterol, urea, and creatinine, with no significant differences among groups. Serum total protein increased (*p* < 0.05) in broiler chickens fed with the C + M, C, and M diets compared to those fed with the CON, AB, and CICC diets. The serum concentration of albumin in the experimental groups did not show any significant changes.

Similar to day 21, serum total triglyceride, cholesterol, urea, and creatinine levels in the three *B. amyloliquefaciens* LFB112 groups were lower (*p* > 0.05) than those in the control group at day 39, but the differences were not significant. The contents of serum glucose and total protein in the C + M, C, and M groups were significantly high compared to the CON. However, they did not show any significant changes across the experimental groups. Supplementation of AB increased serum urea level by 34.0%, 20.4%, 11.3%, 40.5%, and 43.9% compared with the CON, C + M, C, M, and CICC groups respectively.

## 3. Discussion

Previous studies have reported that probiotic supplementation could improve growth performance and feed utilization in chickens [33,34]. Probiotics have been reported to improve the intestinal epithelium, microbiota, digestion, immune system, and resilience in broiler chickens [35,36]. Feeding probiotics, e.g., *B. subtilis*, might lessen stress-caused dysbiosis, resulting in improved gut functioning and nutrient digestibility.

In the present study, we observed that the body weight of broilers fed with *Bacillus* *amyloliquefaciens* LFB112 or its metabolites was higher than the control and antibiotic groups, especially at day 39, while the daily gain was significantly increased, and the FCR was decreased. The average daily gain of birds fed with *Bacillus* *amyloliquefaciens* LFB112 and its metabolites increased by 13.4%, 13.2%, and 11.3% and by 5.3%, 5.2%, and 3.3% compared with the control and antibiotic groups, respectively. The feed conversion ratio of C + M and AB was decreased by 4.7% and 2.9% compared with the control group, respectively. These data indicate that the addition of *Bacillus subtilis* LFB112 to the diet can promote weight gain and reduce the feed conversion ratio of broilers as effectively as aureomycin. Non-nutritive feed additives, such as probiotics containing *Bacillus* spores, can enhance feed utilization, seen as a lower feed conversion ratio [37]. The differences in final body weights of our results may have originated from different feed consumption. The current outcomes were consistent with the study performed by Zhang et al. [38,39], who reported that the administration of 10^5^ and 10^8^ CFU/kg of *Bacillus*-based probiotic enhanced BWG. Other studies showed that dietary supplementation with multiple strains of *Bacillus* or *B. coagulans* significantly improved BWG, ADG, ADFI, and FCR of broilers at days 21–42 and days 1–42 [10,40,41]. In our previous study, Wei et al. [32] showed that dietary supplementation of *B. amyloliquefaciens* LFB112 in 10^8^ to 10^9^ CFU/kg enhanced ADG and ADFI broilers. In contrast, Zeng et al. [42] reported that compound probiotic supplementation significantly increased the ADG in birds from days 1–42 but resulted in no dramatic change in ADG at the early growth stages. Their explanation indicated that the growth-promoting effects of these probiotics mainly support later growth stages. Various studies reported that probiotics have no substantial effect on body weight gain [43,44]. In our experiments, the outcomes were thought to be induced by the probiotic supplementation, comprising gut microbiota regulation, elevated digestion, and enhanced intestinal enzyme activities. However, there are some incongruous results due to differences in probiotic strains, administration dosage, preparation method, bird age, feed composition, hygienic state, and dietary probiotics, which had low or no effects on the growth performance of broiler chickens [36,39,45]. Administration of *B. subtilis* as a direct-fed microorganism (DFM) improved BW, BWG, and ADFI in comparison to those fed with untreated diet and to those administered an antibiotic growth promoter (AGP), suggesting that DFM administration may be used as an alternative to AGP [46].

The carcass yield, semi-eviscerated rate, and eviscerated rate in carcass characteristics are the main indicators to evaluate the meat performance of broiler chickens. The muscles of broilers are mainly produced in the breast and legs, and their yield and quality are directly related to the meat production performance of broilers. In our study, dietary *Bacillus* *amyloliquefaciens* LFB112 significantly increased the carcass yield, semi-eviscerated rate, eviscerated rate, thigh muscle, and breast muscle weight compared to the control and other groups of broilers chickens. Administration of *B. subtilis* as a direct-fed microorganism (DFM) demonstrated improvements in body weight and breast meat percentage compared to a non-DFM-treated control group [47]. Accordingly, higher carcass yield and individual meat cuts were found in animals fed *B. subtilis*-supplemented feed compared with the control [48,49]. On the contrary, Sarangi et al. [50] found that the percentage of carcass yield did not show any significant increase upon probiotic inclusion compared to the control in broiler chickens.

The abdominal fat represents the main fat deposition in broiler chicken and is likely to be directly related to total fat. Excessive accumulation of abdominal fat indicates not only processing and waste problems but also unproductive feed energy use. A study showed that chickens fed with *B. coagulans* supplemental had lower abdominal fat and higher leg weight than the non-fed *B. coagulans* groups [41]. Santoso et al. [51] found that abdominal fat contents were reduced in broilers supplemented with *B. subtilis* at day 42. Weis et al. also reported significantly less abdominal fat in Ross 308 broiler chickens supplemented with *S. faecium* [52]. In our study, the abdominal fat rate was lower in birds fed with *B.* *amyloliquefaciens* LFB112 or with its cell-free metabolites than in birds fed with a normal diet. Various other studies also showed that *Bacillus subtilis* or other probiotics have beneficial effects on the gizzard, liver, spleen, breast, thigh, heart, and abdominal fat [44,53,54]. As a green feed additive, *B. subtilis* has been widely promoted as an alternative to replace in-feed antibiotics due to its ability to improve livestock production and efficiency [55]. Our results showed that probiotic additives containing *B. amyloliquefaciens* LFB112 and its metabolites were more effective in elevating the live weight and carcass yields than *B. subtilis* fed CICC and antibiotics.

The immune organ index is an important indicator to measure cellular and humoral immunity. The thymus is the central organ of cellular immunity. The bursa of Fabricius is a humoral immune organ specific to poultry. The spleen is the largest peripheral immune organ of poultry, which participates in the cellular and humoral immunity of the whole body. Therefore, the immune status of chickens was evaluated by measuring the weight of immune organs such as the bursa of Fabricius, spleen, and thymus. Probiotics based on *Bacillus* species have been reported to regulate the immune system in broilers. Previous studies have shown that dietary supplementation with *B. subtilis* significantly augmented the size of broiler spleen [39] and bursa [56]. Luan et al. [57] found that dietary spraying with *B. amyloliquefaciens* enlarged the spleen and bursa in the broilers. The results by Soliman et al. showed that supplementation of 1.5 and 2.0 g *B. subtilis* (5 × 10^6^ CFU/g in each liter of drinking water) led to increased carcass, edible organ, and immune organ weights [58]. These reports were consistent with our finding that the addition of *Bacillus* *amyloliquefaciens* LFB112 (C + M) could significantly improve the thymus and spleen index of broilers. The addition of LFB112 bacteria cells alone markedly increased the thymus and spleen index of 21 day broilers. *Bacillus* *amyloliquefaciens* is thought to have stimulated B cells in the spleen, enhancing the immune system through immunoglobulin synthesis. Correspondingly, certain synbiotics may increase the expression of IL-4 and IL-6 genes in the parenchyma of the spleen, which then stimulates B cells [59]. We hypothesized that this result might be related to *Bacillus* *amyloliquefaciens* LFB112 bacterial cells and its cell-free metabolites having a better effect on the upregulation of related gene expression in the spleen. These findings are similar to those of Awad et al. [54], who reported that spleen weight was higher in the probiotic-supplemented group. There was a trend of increase in thymus relative weight on days 1, 7, and 35 of birds after in ovo inoculation of prebiotics and synbiotics [60]. Interestingly, the relative weights of thymus and bursa decreased in the group infected with *S. gallinarum* but increased after being treated orally with the probiotic *B. subtilis* A [61]. Shabani et al. explained that this enhanced weight might be due to a compensatory mechanism through which more antibodies were produced to combat the disease condition [62]. However, Sikandar et al. observed no significant differences between untreated groups in bursa weight compared to higher weights observed in *Bacillus*-supplemented groups on day 35 [63].

Serum immunoglobulins, particularly IgA, IgG (its avian correspondent, IgY), and IgM generated by B cells, serve as key indicators representing an animal’s humoral immunological state, owing to their vital functions in immune regulation and resistance to various ailments [64,65]. Previous studies examining the effect of probiotics showed that dietary supplementation with *B. subtilis* or *B. amyloliquefaciens* modified the immune response in broilers and mice, whereby serum IgG, IgM, and IgA showed increased and diverse levels [57,66,67]. In the current study, *B.* *amyloliquefaciens* LFB112 substantially upregulated IgA and IgG concentrations in the serum of broilers on days 21 and 39, indicating an improvement in immune function. The results of this study are consistent with the research in which supplementation of *Bacillus subtilis* DSM 29,784 significantly upregulated serum IgA and IgG contents compared to the control or 250 mg/kg enramycin group [68]. Another report demonstrated that broilers receiving *Saccharomyces boulardii* and *Bacillus subtilis* B10 (1 × 10^8^ CFU/kg of feed) significantly increased the number of IgA-positive cells in the jejunum, as well as the cytokine IL-6, TNF-α, TGF-β, and sIgA concentration levels in both jejunum and ileum [11]. Unfortunately, we did not measure the levels of cytokines in the serum or intestine. However, various experiments have shown that dietary probiotics pretreatments enhanced the humoral immune responses in birds by improving the content of immunoglobulins [66,69]. Yisa et al. [70] and Awais et al. [71] concluded that the inclusion of 1 g of probiotics in the diet can stimulate the immune system and the proliferation of beneficial microorganisms in the gut. Moreover, the enhancement of broiler gut mucosal immunity by feeding a diet supplemented with probiotics can be attributed to the ability of probiotics to increase the levels of secretory IgA [72]. The relative size of the immune organs is directly related to their immune function [73]. In the present study, the results of the relative weight of the immune organs were improved at both the starter and the grower phase. These results are consistent with those of the serum immune factor in terms of IgA, IgG, and IgM, which was higher in birds administered LFB112 treatments compared to the control from day 1 to 39. These results indicate that adding *B. amyloliquefaciens* LFB112 and its metabolites positively affected the development of the immune system of Ross 308 broilers between days 1 and 39.

Serum biochemical indices can partly reflect the metabolism and health status of the organism, especially serum total protein, albumin, and urea nitrogen, which reflect the metabolic status of protein in the body to a certain extent. Glucose is an important cellular source of energy and serves as a metabolic substrate, which reflects the physiological state of animals. In some manner, glucose content is positively correlated with growth rate. In the present study, serum glucose and total protein were higher in C + M, C, and M groups than in the control group on days 21 and 39. Our results are consistent with the observations of Shatskikh et al. [74], who noted a higher content of glucose in blood serum in chickens fed with additive “ProStor”, which includes spore-forming *Bacillus subtilis* auxiliary substances, compared to the control. However, in the broilers of the first experimental group, the difference in this indicator with the control was insignificant (lower by 0.3%). Similar findings were also reported by Abudabos et al. [26], who noted that serum glucose and protein increased significantly in broilers challenged with *Salmonella* in response to different feed additives, including *Bacillus subtilis*, *Saccharomyces boulardii,* and oregano. However, Abudabos et al. found no significant influence on blood glucose and protein levels in addition to multiple feed additives, including *B. subtilis*, in *Salmonella*-infected broilers during the starter phase [75]. Hussein et al. [76] showed similar data, with higher glucose levels in probiotic-supplemented chickens. On the contrary, Gong et al. reported that the serum glucose concentration of broilers fed with *B. subtilis natto* decreased compared to the control group [77]. The best explanation for these results might be that the metabolism of broilers gradually improved due to the probiotics promoting the function of animal cells to utilize glucose. In the present study, we infer that *B.* *amyloliquefaciens* LFB112 as a feed additive probably increased the intestine glucose channels, leading to a greater absorption of nutrients, which further increased blood glucose level. On the other hand, the better growth performance and carcass quality may have partially contributed to the higher protein profile and glucose level of supplemented birds.

Serum total protein is an important index to reflect the deposition of protein in animals. Albumin is mainly synthesized by the liver, and it has the functions of transporting metabolites in the body, maintaining colloidal osmotic stability, and protecting globulin in the blood. The serum albumin level of broilers increased linearly with the *Bacillus subtilis* supplementation dosage. Serum albumin and globulin are two major components of total protein and important indicators of hepatic damage and function. In this study, we found that dietary supplementation of *Bacillus* *amyloliquefaciens* LFB112 and its metabolites could increase the contents of serum total protein and albumin throughout the feeding process. The increase in serum total protein and albumin could be explained by the inhibition exclusion mechanism, in which *B. subtilis* improves dietary protein utilization by inhibiting pathogen growth via the secretion of antibacterial substances, reducing protein breakdown into nitrogen, and decreasing dietary protein efficiency, while also increasing the surface area for nutrient absorption [78]. Our previous study showed that the secretary substances of LFB112 have inhibitory activity toward a wide range of pathogens, including some important animal pathogens and two multidrug-resistant clinical isolates of *Escherichia coli* and *Salmonella pullorum*, as well as a phytopathogenic yeast strain [30]. We suggest that LFB112 may improve serum total protein and immunoglobulin by enhancing mucosal immunity and nutrition absorption. Another explanation could be related to one of the primary routes of action of probiotics being immune system stimulation. Plasma immunoglobulin concentrations can be used to assess an animal’s humoral immunological status. Good nutritional status can maintain the contents of serum total protein and albumin at a high level, and their increase indicates that the body’s metabolic activity is vigorous [79]. Serum total protein content is an effective indicator of protein metabolism in poultry, whereby a higher serum total protein content indicates more vigorous protein metabolism and good nutritional performance in poultry [80]. However, no significant difference in serum total protein, albumin, or globulin was demonstrated in chicks fed diets supplemented with or without probiotics including *B. subtilis* and *B. licheniformis* [81].

The ammonia produced by poultry metabolism mainly leads to the synthesis of urea through the liver and is excreted with urine through the kidney. Urea is the final product of protein metabolism in vivo and is one of the indices to evaluate renal function. We also found that the addition of *Bacillus* *amyloliquefaciens* LFB112 and its metabolites reduced serum urea and creatinine levels. These results indicate that the addition of *Bacillus* *amyloliquefaciens* LFB112 to the diet not only improves the ability of protein synthesis and promotes protein deposition in broilers, but also alleviates the pressure of the kidney and reduces the content of serum urea. In line with our findings, many studies also reported that the addition of probiotics decreased nonprotein nitrogen in chicken blood, including uric acid, ammonia, and urea [82,83].

The concentration of lipid components such as cholesterol and triglycerides in chicken blood serum was used to measure lipid metabolism. Cholesterol is an important biomolecule since it is the building block of cell membranes and bile, a precursor of vitamin D and many hormones, which are also found in all cells of living organisms, being necessary for development. In our studies, the cholesterol level in birds fed with *B.* *amyloliquefaciens* LFB112 and its metabolites was lower than the control group at days 21 and 39, although no significant difference was observed. It has been revealed that the enzymatic conversion of cholesterol to coprostanol by probiotics in the intestines promotes their excretion through feces. In a homeostatic reaction, this removal transfers more cholesterol to the creation of new bile acids, resulting in a decrease in serum cholesterol [84]. Concerning the content of triglycerides, a tendency of their increase in the starter and grower phases was established in chickens of the C + M and C groups compared to the control, which indicates the activation of lipid metabolism in the body of the birds. The above results are consistent with the studies of other authors on broilers that reported lowering of total serum [85] and carcass cholesterol [51] with different probiotic microorganisms. Low cholesterol concentrations in our study could be explained by a study performed by Fukushima and Nakano [86], who suggested that probiotics could also influence the cholesterol blood levels by inhibiting cholesterol synthesis. On the other hand, animals fasting for 8 h before being slaughtered resulted in lower total serum cholesterol [87]. Another reason could be that probiotics lower cholesterol by adhering cholesterol to the cellular membrane of bacterial cells and by deconjugating bile salts, which could disrupt the enterohepatic cycle [88]. In line with the current findings, Hussein et al. reported that, regardless of whether yeast and *Bacillus subtilis* were added alone or mixed in the diet, the contents of total lipids and cholesterol in the serum of broilers were significantly reduced [89]. The previous finding stated that the administration of *B. subtilis natto, B. licheniformis,* and *B. cereus* markedly reduced ammonia, uric acid level, total cholesterol, and triglyceride in serum [77]. Similar to Shatskikh et al. [74], additive “ProStor” including spore-forming *Bacillus subtilis* auxiliary substances in feed lowered cholesterol levels compared to control broiler chickens. However, Saleh et al. revealed that feeding broilers on *B. licheniformis*-fermented products did not affect their blood concentrations of glucose, triglycerides, and total cholesterol [90]. In our observation, we noted that, against the background of the use of supplements containing LFB112 or its metabolites, an improvement in the assimilation of protein nitrogen was observed in the experimental groups, as evidenced by an increase in total protein and a decrease in urea and creatinine in the blood serum. Furthermore, the administration of the investigated probiotic was associated with the activation of lipid metabolism in the body of birds, as defined by a propensity to lower the quantity of total cholesterol and triglycerides in blood serum at day 21 and day 39.

## 4. Materials and Methods

The protocol was reviewed and approved by the Animal Care and Use Committee of China Agricultural University (CARE NO.AW17109102-1-1). The experiment was performed at the DaYong broiler-breeding corporation (chicken farm) located in Henan province, China. All procedures were performed strictly following the guidelines of recommendations in the Guide for Experimental Animals of the Ministry of Science and Technology (Beijing, China), and maximum efforts were made to minimize suffering.

### 4.1. Preparation of Bacterial Strain Powder

*Bacillus amyloliquefaciens* LFB112 was isolated from Chinese herbs using brain heart infusion (BHI) medium. The isolate was identified to a species level according to the profiles of API tests and 16S rDNA sequence analysis, and it was assigned GenBank accession number FJ527490 [30]. The strain was deposited in the China General Microbiological Culture Collection Center (CGMCC No. 2996). The LFB112 strain was cultured in Landy medium for 24 h at 30 °C, and then inoculated in a 50 L vertical fermentation tank (GuJS-50, Zhenjiang Dongfang Bioengineering Technology Co., LTD., Zhenjiang, China) at 5% for 20 h. Then, the fermentation suspensions were directly spray-dried with maltodextrin as a carrier to form a powder which included vegetative cells and metabolites.

Metabolites were removed from *B. amyloliquefaciens* LFB112 fermentation suspensions by centrifugation at 3000× *g* for 10 min, before being washed and resolubilized in PBS; then, a pure vegetative cell powder was obtained by spray-drying. Bacterial cells were removed from *B. amyloliquefaciens* LFB112 fermentation liquor by ceramic membrane filtration, and a cell-free supernatant powder was formed by spray-drying. Colony-forming units (CFU) were determined using the plate counting method. The fermentation suspensions were tenfold diluted in series to 10^5^ CFU/mL, 10^6^ CFU/mL, and 10^7^ CFU/mL, before pouring 100 µL of the dilution into a Landy medium agar plate with three duplicates and incubating at 37 °C for 24 h before counting. The contents of strain powder (vegetative cells + metabolites), vegetative cell powder, and cell-free supernatant powder were 10^11^ CFU/g. Then, 5 × 10^8^ CFU/kg was added to the feed.

The standard strain of *Bacillus subtilis* CICC 20,179 was used as the negative control, provided by the China Center of Industrial Culture Collection (CICC). Its cultivation, fermentation, and powder processing were as described for *B. amyloliquefaciens* LFB112. The concentration of *Bacillus subtilis* CICC 20,179 after fermentation was 10^11^ CFU/g, and 5 × 10^8^ CFU/kg was added to dietary feed.

### 4.2. Birds, Diets, and Management

A total of 396 1 day old commercial Ross 308 broilers of mixed sexes with similar body weight were assigned to six experimental groups, each including six replicates with 11 birds per replicate. The chickens were kept in metal mesh cages equipped with nipple drinkers and feeders. The chickens in each group were evenly divided into two cages on days 1–9 and six cages after day 10. The ambient temperature for the first day was maintained at 33 °C, reduced by 0.5 °C until the seventh day, and by 0.3 °C from the eighth day to the 30th day, until it reached 22 °C. The relative humidity was maintained at 60–70% from day 1 to day 14 and at 50% afterward. A continuous lighting program was adopted throughout the entire experimental period. Mash feed and freshwater were provided ad libitum. The dietary information is given in Table 4. All nutrients met or exceeded the NRC (1994) recommendations. The six dietary treatments were formulated as follows: CON group (basal diet with no supplement), AB (antibiotics) group (basal diet + 150 mg of aureomycin/kg), C + M group (basal diet + 5 × 10^8^ CFU/kg *B. amyloliquefaciens* LFB112 powder with vegetative cells + metabolites); C group (basal diet + 5 × 10^8^ CFU/kg *B. amyloliquefaciens* LFB112 vegetative cell powder with removed metabolites); M group (basal diet + 5 × 10^8^ CFU/kg *B. amyloliquefaciens* LFB112 metabolite powder with removed vegetative cells); CICC group (basal diet + 5 × 10^8^ CFU/Kg *Bacillus* *subtilis* CICC 20179). The experiment lasted 39 days with two feeding periods. The starter period was from day 1 to 21, and the finisher period was from day 22 to 39.

### 4.3. Sampling and Measurements

The initial live weights of the birds were measured at the beginning of the experiment. The body weight and feed intake of each bird were recorded weekly. Feed intake and BW were recorded on days 21 and 39 for each pen after a 12 h feed withdrawal. The average daily gain (ADG), average daily feed intake (ADFI), and feed conversion ratio (FCR) was computed for each group using the following formula: FCR = feed intake/weight gain [91]. Mortality was recorded daily, and ADG, ADFI, and FCR were corrected by mortality.

On days 21 and 39 of the experiment, one bird from each pen (11 birds per treatment) with body weight (BW) close to the average was selected and slaughtered. Feed was restricted 12 h before slaughter. Blood was drawn from the wing vein with vacuum blood collection tubes and centrifuged at 3000× *g* for 15 min to obtain serum. The serum was stored at −20 °C immediately for the detection of immunoglobulins and biochemical parameters. The contents of serum immunoglobulins (IgA, IgM, and IgG) were tested using specific ELISA kits (Nanjing Jiancheng Bioengineering Institute, Nanjing, China). Serum glucose (GLU), total cholesterol (TC), triglyceride (TG), total protein (TP), albumin (ALB), urea, and creatinine were analyzed using ELISA kits (Cusabio Biotech Co., Ltd., Hubei, China) following the manufacturer’s instructions.

On day 39, broilers of average body weight from each replicate were chosen, weighed live, and then slaughtered. Carcass weight was obtained after removal of blood and feathers. Semi-eviscerated weight was chilled carcass weight minus that of the trachea, esophagus, crop, intestine, spleen, pancreas, gonads, gallbladder, contents of the proventriculus, and gizzard lining. Eviscerated weight was the semi-eviscerated carcass weight minus that of the head, neck, shank, heart, liver, gizzard, proventriculus, and abdominal fat. Carcass yield (%) = carcass weight/live weight × 100; eviscerated rate (%) = eviscerated weight/live weight × 100; semi-eviscerated rate (%) = semi-eviscerated weight/live weight × 100; breast muscle yield (%) = ambilateral breast muscle weight/eviscerated weight × 100; thigh muscle yield (%) = ambilateral thigh muscle weight/eviscerated weight × 100; abdominal fat rate (%) = abdominal fat weight/(eviscerated weight + abdominal fat weight). The thymus, spleen, and bursa of Fabricius were dissected and removed. Individual immune organ weights were recorded and expressed relative to BW (g of organ/kg of BW).

### 4.4. Statistical Analysis

The pen was employed as the experimental unit, and the data’s homogeneity and normality were checked first. The data were subjected to one-way ANOVA in the GLM using the statistical package of SPSS version 20.0 for Windows (SPSS Inc., Chicago, IL, USA). The results were expressed as means with their standard error of the mean (SEM). Duncan’s multiple-comparison test was used to identify the major differences between treatments, and a *t*-test was performed to define the statistically significant results (*p* < 0.05).

## 5. Conclusions

*Bacillus amyloliquefaciens* LFB112 or its metabolites improve the ability of protein synthesis, as well as promote protein deposition and glucose utilization in broilers. Results show that the growth performance, feed conversion ratio, carcass yield, breast and leg meat, and immunity of broiler birds can be significantly improved by dietary inclusion of *Bacillus amyloliquefaciens* LFB112 or its metabolites. These results suggest that *B. amyloliquefaciens* LFB112 can be used to a great extent as a potential alternative to antibiotic growth promoters in broiler nutrition. According to our results, broiler diets can be supplemented with 5 × 10^8^ CFU/kg *Bacillus amyloliquefaciens* LFB112 powder to promote growth performance, carcass yield, and immunity.

## Figures and Tables

**Table 1:** Effect of *Bacillus amyloliquefaciens* LFB112 on growth performance of broilers ^1^.

Item	Treatments ^2^						SEM	*p*-Value
	CON	AB	C + M	C	M	CICC		
Initial BW (g)	47.67	47.35	47.68	47.55	47.40	47.23	0.040	0.901
1 to 21 days								
ADG (g/day)	23.94 ^c^	23.53b ^c^	25.93 ^ab^	24.67 ^ab^	26.00 ^a^	25.36 ^a^	0.138	0.028
ADFI (g/day)	37.82	36.43	37.97	37.74	39.78	40.32	0.251	0.052
FCR	1.58 ^ab^	1.55 ^ab^	1.58 ^ab^	1.53 ^b^	1.57 ^ab^	1.59 ^a^	0.006	0.014
22 to 39 days								
ADG (g/day)	60.61 ^c^	62.27 ^bc^	66.89 ^ab^	68.90 ^a^	67.09 ^ab^	61.53 ^c^	0.940	0.003
ADFI (g/day)	106.07 ^b^	110.88 ^ab^	113.71 ^a^	118.87 ^a^	116.76 ^a^	106.08 ^b^	1.540	0.006
FCR	1.75 ^a^	1.78 ^a^	1.70 ^b^	1.73 ^ab^	1.74 ^ab^	1.73 ^ab^	0.014	0.020
1 to 39 days								
ADG (g/day)	40.33 ^b^	43.41 ^b^	45.72 ^a^	45.65 ^a^	44.89 ^a^	41.73 ^b^	0.462	<0.001
ADFI (g/day)	66.43 ^bc^	70.83 ^b^	73.23 ^ab^	75.31 ^a^	73.70 ^ab^	68.25 ^b^	0.905	0.020
FCR	1.68 ^a^	1.63 ^ab^	1.60 ^b^	1.63 ^a^	1.64 ^a^	1.64 ^a^	0.011	0.011
Final BW (g)	1620.17 ^b^	1662.37 ^b^	1830.58 ^a^	1821.21 ^a^	1798.21 ^a^	1675.04 ^b^	18.901	<0.001
Total gain weight (g)	1572.37 ^b^	1614.77 ^b^	1783.28 ^a^	1773.31 ^a^	1750.61 ^a^	1627.44 ^b^	15.182	<0.001

SEM = standard error of the mean. ^a–c^ Different superscript letters within a row denote a significant difference (*p* < 0.05). ^1^ Each value represents the mean of six replicates of 11 birds per cage. ^2^ The experimental groups assigned were as follows: CON = basal diet; AB (antibiotics) = basal diet + aureomycin 150 mg/kg; C + M = basal diet + LFB112 fermentation dry powder with *Bacillus* cells + metabolites (5 × 10^8^ CFU/g); C = basal diet + LFB112 *Bacillus* cell powder with removed metabolites (5 × 10^8^ CFU/g); M = basal diet + LFB112 metabolite powder with removed *Bacillus* cells (5 × 10^8^ CFU/g); CICC = basal diet + *Bacillus subtilis* 20,179 (5 × 10^8^ CFU/g).

**Table 2:** Effects of *Bacillus amyloliquefaciens* LFB112 on carcass characteristics of broilers at 39 days of age (%) ^1,2^.

Item	Treatment ^3^						SEM	*p*-Value
	CON	AB	C + M	C	M	CICC		
Carcass yield	88.23 ^b^	89.35 ^ab^	91.77 ^a^	91.86 ^a^	90.32 ^ab^	89.25 ^ab^	0.354	0.006
Semi-eviscerated rate	80.16 ^b^	81.03 ^b^	86.21 ^a^	83.18 ^ab^	81.69 ^ab^	81.22 ^b^	0.526	0.005
Eviscerated rate	68.30 ^b^	68.90 ^b^	73.99 ^a^	69.24 ^b^	69.15 ^b^	68.69 ^b^	0.471	0.001
Thigh muscle yield	12.97 ^ab^	13.61 ^ab^	14.67 ^a^	14.20 ^ab^	13.92 ^ab^	12.68 ^b^	0.192	0.015
Breast muscle yield	14.37 ^b^	14.81 ^ab^	16.41 ^a^	15.48 ^ab^	15.44 ^ab^	13.85 ^b^	0.227	0.010
Abdominal fat	1.72 ^a^	1.37 ^bc^	1.21 ^bc^	1.34 ^bc^	1.38 ^ab^	1.48 ^c^	0.050	0.012

SEM = standard error of the mean. ^1^ Carcass phenotypic data were recorded as means of six pens with four sacrificed broilers per pen. ^2^ In all considered parameters, the skin and bone were removed when calculating the BW at slaughter. ^3^ Dietary treatments were as follows: CON = basal diet; AB (antibiotics) = basal diet + aureomycin 150 mg/kg; C + M = basal diet + LFB112 fermentation dry powder with *Bacillus* cells + metabolites (5 × 10^8^ CFU/g); C = basal diet + LFB112 *Bacillus* cell powder with removed metabolites (5 × 10^8^ CFU/g); M = basal diet + LFB112 metabolite powder with removed *Bacillus* cells (5 × 10^8^ CFU/g); CICC = basal diet + *Bacillus subtilis* 20,179 (5 × 10^8^ CFU/g). ^a–c^ Different superscript letters within a row denote a significant difference (*p* < 0.05).

**Table 3:** Effect of *Bacillus* *amyloliquefaciens* LFB112 on serum biochemical indices of broilers.

Item	Treatment ^1^						SEM	*p*-Value
	CON	AB	C + M	C	M	CICC		
21 d								
Glucose (mmol/L)	10.67 ^ab^	10.98 ^ab^	11.31 ^a^	11.21 ^a^	11.01 ^ab^	9.45 ^b^	0.175	0.011
Total cholesterol (mmol/L)	3.34 ^ab^	4.12 ^a^	3.31 ^b^	3.49 ^ab^	3.05 ^b^	3.35 ^ab^	0.090	0.008
Triglyceride (mmol/L)	0.32 ^b^	0.39 ^b^	0.53 ^a^	0.50 ^a^	0.49 ^a^	0.41 ^b^	0.015	<0.001
Total protein (g/L)	25.50 ^b^	23.12 ^c^	26.23 ^a^	27.32 ^a^	25.73 ^ab^	25.57 ^b^	0.367	0.002
Albumin (g/L)	12.00	10.65	10.83	11.70	11.15	10.98	0.181	0.221
Urea (mmol/L)	0.53 ^ab^	0.59 ^a^	0.44 ^b^	0.49 ^ab^	0.44 ^b^	0.51 ^ab^	0.017	0.049
Creatinine (μmol/L)	9.05 ^b^	11.92 ^a^	10.33 ^b^	9.6 ^b^	8.78 ^b^	9.53 ^b^	0.266	0.003
39 d								
Glucose (mmol/L)	10.50	11.09	12.06	11.13	10.82	10.77	0.161	0.079
Total cholesterol (mmol/L)	3.57	2.95	3.22	3.02	3.07	3.76	0.108	0.176
Triglyceride (mmol/L)	0.37 ^b^	0.45 ^b^	0.52 ^a^	0.56 ^a^	0.43 ^b^	0.42 ^b^	0.016	0.004
Total protein (g/L)	31.85	27.15	32.63	32.85	34.00	34.48	0.840	0.117
Albumin (g/L)	10.38 ^b^	9.48 ^b^	11.92 ^a^	11.15 ^a^	11.38 ^a^	11.20 ^a^	0.170	0.003
Urea (mmol/L)	0.44 ^b^	0.59 ^a^	0.49 ^ab^	0.53 ^ab^	0.41 ^b^	0.42 ^b^	0.018	0.017
Creatinine (μmol/L)	9.75 ^bc^	11.43 ^ab^	10.23 ^ab^	11.75 ^a^	8.28 ^c^	9.62 ^bc^	0.305	0.005

SEM = standard error of the mean. ^a–c^ Different superscript letters within a row denote a significant difference (*p* < 0.05). ^1^ Number of replicates per group (*n* = 6). AB (antibiotics) = basal diet + aureomycin 150 mg/kg; C + M = basal diet + LFB112 fermentation dry powder with *Bacillus* cells + metabolites (5 × 10^8^ CFU/g); C = basal diet + LFB112 *Bacillus* cell powder with removed metabolites (5 × 10^8^ CFU/g); M = basal diet + LFB112 metabolite powder with removed *Bacillus* cells (5 × 10^8^ CFU/g); CICC = basal diet + *Bacillus subtilis* 20,179 (5 × 10^8^ CFU/g).

**Table 4:** Ingredient and nutrient composition of the basal diet (as-fed basis, % unless noted).

Item	Starter (1–21 Days)	Finisher (22–39 Days)
Ingredients (%)		
Corn	54.70	56.90
Soybean meal	34.70	32.40
Dicalcium phosphate	1.50	1.40
Limestone	1.20	1.20
Soybean oil	0.90	1.10
Wheat	5.00	5.00
Chicken bone meal	0.00	2.00
Premix ^1^	2.00	2.00
Total	100.00	100.00
Nutrient levels ^2^		
ME (kcal/kg)	3100.00	3200.00
Dry matter (%)	87.35	87.70
Crude protein (%)	22.00	20.50
Crude fiber (%)	3.40	3.50
Lysine (%)	1.40	1.27
Methionine + cystine (%)	0.98	0.93
Threonine (%)	0.95	0.84
Calcium (%)	0.93	0.90
Total phosphorus (%)	0.69	0.66

^1^ The premix diet provides the following per kilogram: vitamin A, 14,000 IU; vitamin D3, 6000 IU; vitamin E, 70 mg; vitamin K3, 4 mg; vitamin B1, 7 mg; vitamin B2, 13 mg; vitamin B6, 13 mg; vitamin B12, 29 μg; choline, 1835 mg; folic acid, 3 mg; nicotinic acid, 93 mg; pantothenic acid, 27 mg; Fe, 111 mg; Cu, 10 mg; Mn, 128 mg; Zn, 142 mg. ^2^ Nutrient levels were calculated values.

## Data Availability

Data sharing is not applicable to this article.

## Supplementary Materials

The following are available online at https://www.mdpi.com/article/10.3390/antibiotics10111427/s1, Table S1. Effect of *Bacillus amyloliquefaciens* LFB112 on immune organ index (g/kg) of broilers. Table S2. Effect of *Bacillus amyloliquefaciens* LFB112 on serum immune factors (g/L) of broiler.
