# Supplementary material for: Effects of Bacillus amyloliquefaciens LFB112 on Growth Performance, Carcass Traits, Immune, and Serum Biochemical Response in Broiler Chickens
Source: Antibiotics (Basel). 2021 Nov 22;10(11):1427. doi: 10.3390/antibiotics10111427 (PMC8614806; doi:10.3390/antibiotics10111427)
Supplement: Supplementary file 1 [file antibiotics-10-01427-s001.zip › antibiotics-1451939-supplementary.pdf]

antibiotics

Supplementary

Effects of *Bacillus amyloliquefaciens* LFB112 on Growth Performance, Carcass Traits, Immune, and Serum Biochemical Response in Broiler Chickens

Marhaba Ahmat [1,2], Junhao Cheng [1], Zaheer Abbas [1], Qiang Cheng [1], Zhen Fan [3], Baseer Ahmad [4], Min Hou [2], Ghenijan Osman [2], Henan Guo [1], Junyong Wang [1] and Rijun Zhang [1,*]

[1] Laboratory of Feed Biotechnology, State Key Laboratory of Animal Nutrition, College of Animal Science and Technology, China Agricultural University, Beijing 100193, China; malika511@126.com (M.A.); chengjunhao@cau.edu.cn (J.C.); Zaheerabbas113@yahoo.com (Z.A.); chengqiangcool@163.com (Q.C.); ghn_657@cau.edu.cn (H.G.); wangjy9722@163.com (J.W.)
[2] Xinjiang Laboratory of Special Environmental Microbiology, Institute of Applied Microbiology, Xinjiang Academy of Agricultural Sciences, Urumqi 830091, China; hmde_092@163.com (M.H.) ghenijan85@foxmail.com (G.O.)
[3] TECON Biology Company, LTD 528 Changchun South Road, Urumqi 830091, China; fanzhen1222@outlook.com
[4] Muhammad Nawaz Sharif University of Agriculture, Multan 25000, Pakistan; dr.baseerahmadkhan@gmail.com
* Correspondence: zhangrj621@126.com

Table S1. Effect of *Bacillus amyloliquefaciens* LFB112 on immune organ index (g/kg) of broilers.[1]

Item	Treatment[2]						SEM	*P*-value
	CON	AB	C+M	C	M	CICC		
				21 d				
Thymus	2.42c	2.75abc	3.17a	3.02ab	2.35c	2.50bc	0.009	0.032
Bursa of Fabricius	2.48bc	2.68ab	2.65ab	2.73ab	2.17c	2.07c	0.008	0.031
Spleen	1.00b	1.28a	1.00b	1.33a	0.77c	0.97bc	0.004	<0.001
				39 d				
Thymus	2.18abc	2.53a	2.50a	2.65a	2.30ab	1.72c	0.008	0.003
Bursa of Fabricius	2.18abc	1.97ab	1.90ab	2.02ab	2.12a	1.60b	0.006	0.041
Spleen	1.10bc	1.32ab	1.48a	0.97c	0.98c	1.07bc	0.005	<0.001

SEM=standard error of the mean. [a–c] Within a row, numbers with different superscripts differ statistically at $P<0.05$. [1] Six replicates per treatment group (n =6).[2] Dietary treatments were as follows: CON= basal diet; AB (Antibiotics)=basal diet +aureomycin150 mg/kg; C+M=basal diet + LFB112 fermentation dry powder with *Bacillus* cell +metabolite (5×10^8 CFU/g); C=basal diet+LFB112 *Bacillus* cells powder with removed metabolite (5×10^8 CFU/g); M=basal diet+LFB112 metabolite powder with removed *Bacillus* cells (5×10^8CFU/g); CICC=basal diet +*Bacillus Subtilis* 20179 (5×10^8 CFU/g).

Table S2. Effect of *Bacillus amyloliquefaciens* LFB112 on serum immune factors (g/L) of broiler [1].

Item	Treatment[2]						SEM	*P*-value
	**CON**	**AB**	**C+M**	**C**	**M**	**CICC**		
				21 d				
IgA	0.97ab	0.89b	1.05a	1.04a	0.96ab	0.86b	0.017	0.002
IgG	6.61bc	5.91c	7.80ab	8.50a	7.01bc	7.23b	0.182	<0.001
IgM	0.90ab	0.76b	0.94ab	0.88ab	1.04a	0.82b	0.023	0.004
				39 d				
IgA	1.02a	0.86b	1.20ab	1.18ab	1.09ab	1.03ab	0.034	0.027
IgG	7.65ab	6.42b	8.24a	8.07a	8.04a	7.35ab	0.150	0.001
IgM	0.95	0.85	0.99	1.07	0.84	0.95	0.026	0.077

SEM=standard error of the mean. Ig = immunoglobulin. [a–c] Different superscripts within a row shows significant difference ($P < 0.05$). [1] Number of replicates per group (n =6). [2] Experimental group assigned were: CON= basal diet; AB (Antibiotics)=basal diet +aureomycin150 mg /kg; C+M=basal diet + LFB112 fermentation dry powder with *Bacillus* cell + metabolite (5×10^8 CFU/g); C=basal diet+LFB112 *Bacillus* cells powder with removed metabolite (5×10^8 CFU/g); M=basal diet+LFB112 metabolite powder with removed *Bacillus* cells (5×10^8 CFU/g); CICC=basal diet+ *Bacillus subtilis* 20179 (5×10^8 CFU/g).
